# Solution of Phosphate of Iron and Quinine

**Published:** 1851-08

**Authors:** 


### MATERIA MEDICA AND THERAPEUTICS.

*Solution of Phosphate of Iron and Quinine.*—I have much pleasure in directing the attention of the profession to the therapeutical employment of a compound, formed of phosphoric acid, pure quinia, and hydrated peroxide of iron—solution of phosphate of quinine and iron. It was devised by me during the past year, and from an extensive trial of it, since that time, I am enabled to recommend it as a remedy likely to prove highly serviceable in those cases indicating the use of such a combination. As much uncertainty exists respecting the chemical relations of phosphoric acid, and the different bases, it is to the therapeutical and not the chemical value of this compound that I attach importance. I shall avail myself of the earliest opportunitiy of making further observations on the subject.—*Dr. Cattell of Braunston, Northamptonshire, in Lancet.*

